# Oxytocin receptor induces mammary tumorigenesis through prolactin/p-STAT5 pathway

**DOI:** 10.1038/s41419-021-03849-8

**Published:** 2021-06-07

**Authors:** Dan Li, Mingjun San, Jing Zhang, Anlan Yang, Wanhua Xie, Yang Chen, Xiaodan Lu, Yuntao Zhang, Mingyue Zhao, Xuechao Feng, Yaowu Zheng

**Affiliations:** 1 Transgenic Research Center, Northeast Normal University, Changchun, Jilin 130024 China; 2 The Precise Medicine Center, Department of Basic Medicine, Shenyang Medical College, Shenyang, Liaoning 110034 China; 3 School of Life Sciences, Xuzhou Medical University, Xuzhou, Jiangsu 221004 China; 4 Institute of Biomedical Sciences, Shanxi University, Taiyuan, Shanxi 030006 China

**Keywords:** Breast cancer, Cancer models

## Abstract

Oxytocin receptor (OXTR) is involved in social behaviors, thermoregulation, and milk ejection, yet little is known about its role in breast cancer. To investigate the role of OXTR in mammary gland development and tumorigenesis, a transgenic mouse model of OXTR overexpression (^*++*^*Oxtr*) was used. Overexpression of OXTR-induced progressive mammary hyperplasia, unexpected milk production, and tumorigenesis in females. OXTR-induced mammary tumors showed ERBB2 upregulation and mixed histological subtypes with predomination of papillary and medullary carcinomas. OXTR overexpression led to an activation of prolactin (PRL)/p-STAT5 pathway and created a microenvironment that promotes mammary-specific tumorigenesis. PRL inhibitor bromocriptine (Br) could mitigate OXTR-driven mammary tumor growth. The study demonstrates *Oxtr* is an oncogene and a potential drug target for HER2-type breast cancer.

## Introduction

Breast cancer is the most common cancer with highest morbidity among females worldwide^1^. It is genetically classified into four subtypes, HER2^**+**^, Luminal A, Luminal B, and triple negative (basal-like subtype)^2^. These subtypes differ significantly in prognosis and responsiveness to various therapeutic options^3^. ERBB2 receptor tyrosine kinase (or HER2) is a family member of epidermal growth factor receptors (EGFRs). Overexpression of HER2 induces tumorigenesis^4^. HER2 is overexpressed in 20–30% of breast tumors^5^ and correlates with poor patient outcome^6^. Lapatinib, a tyrosine kinase inhibitor, mitigates mammary tumor growth by blocking HER2 tyrosine kinase activity^7^. Understanding breast cancer development is critical for effective treatments. Mouse models have been developed to mimic clinic phenotypes^8^. MMTV-PyMT mice exhibit papillary and medullary carcinomas^9^ similar to HER2^+^ breast cancer^10^. *Brca1*^−/−^ and *p53*^−/−^ mice grow basal-like tumors^11,12^.

Mammary gland development depends on ordered hormonal environment of estrogen, prolactin (PRL), and progesterone (P4). PRL binds to prolactin receptor (PRLR) and activates signal transducer and activator of transcription 5 (STAT5). Activated STAT5 (p-STAT5) translocates to nucleus of mammary epithelial cells and upregulates transcription of alveolar development-related genes and genes for milk production^13,14^. Mammary carcinogenesis initiates from abnormal mammary gland development. Aberrant PRL/p-STAT5 signaling induces excessive proliferation and mammary tumorigenesis^15,16^. P4 promotes proliferation of mammary epithelium via secretion of RANKL^17,18^. These hyper-proliferations lead to breast cancer^19,20^.

G-protein coupled receptors (GPCRs) regulate a variety of physiologic functions, ranging from blood pressure control, kidney function, allergic response, hormonal disorders to neurologic diseases^21^. Oxytocin receptor (OXTR), a member of GPCRs, is the receptor for neurotransmitter oxytocin (OXT)^22^ known to regulate sexual and social behaviors, thermoregulation, and milk ejection^23^. OXTR has been found highly expressed in pathological breast, breast carcinomas, neuroblastomas, and astrocytomas^24–26^. OXTR overexpression has been reported in endometrial adenocarcinomas^27^. Connections between breast cancer and OXT/OXTR have been suggested^28^. However, whether and how OXTR regulates mammary gland development and carcinogenesis remains unknown. Our previous study indicates OXTR overexpression disrupts hormonal environment, induces early mammary gland maturation, early involution, and lactation failure^29^. Here we investigated OXTR’s role in mammary tumorigenesis using the ^**++**^*Oxtr* mouse model.

## Results

### OXTR overexpression induces mammary tumorigenesis

**Fig. 1:**
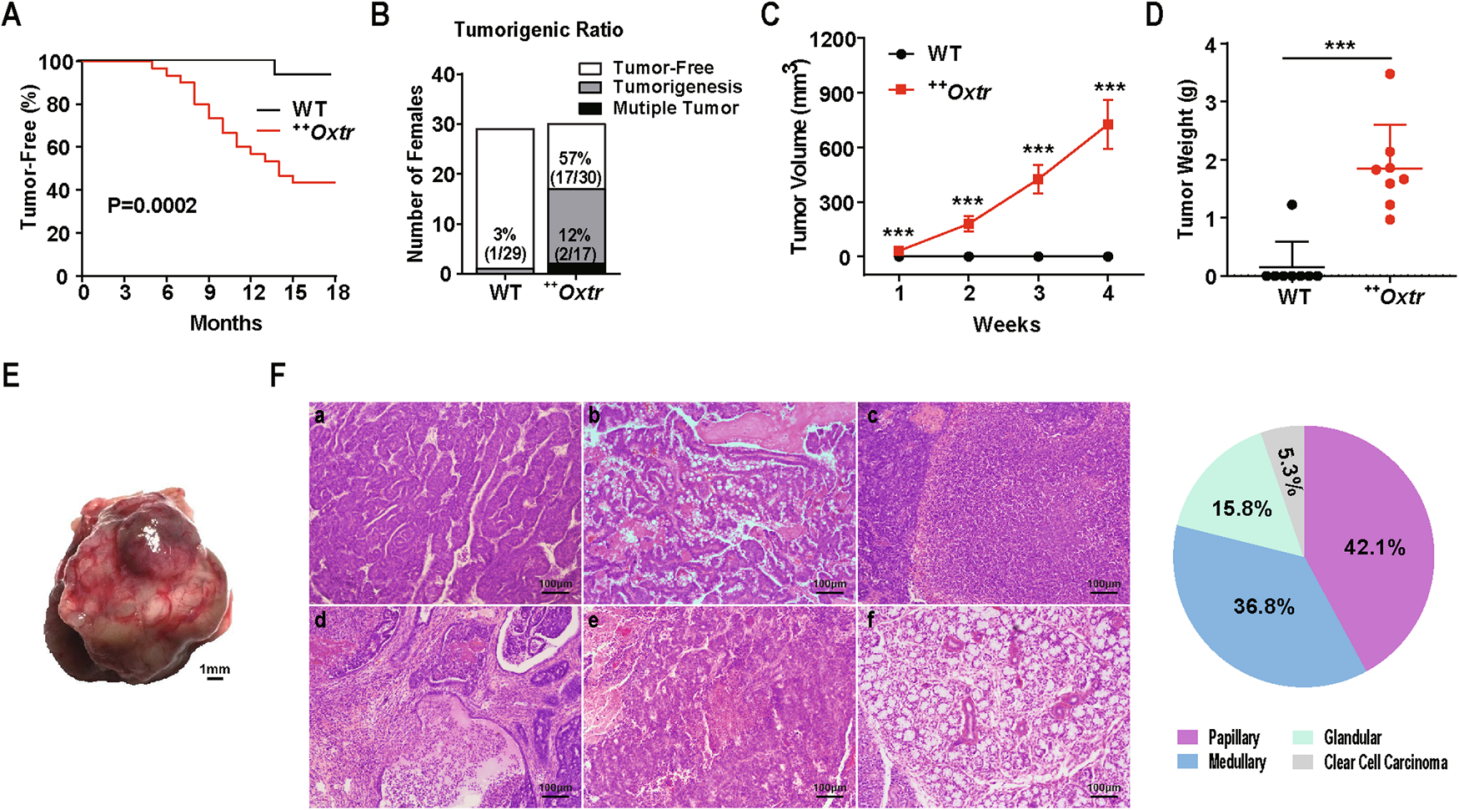
Tumor onset and histology analysis. **A** Kaplan–Meier plot of tumor-free survival for wild-type (WT) (*n* = 29) and ^*++*^*Oxtr* females (*n* = 30). **B** Tumor incidence analysis, WT, *n* = 29 and ^*++*^*Oxtr*, *n* = 30. **C** Tumor growth measurement by volume (*n* = 9). **D** Tumor weights 30 days after palpation (*n* = 8). Data were represented as mean ± SD. ***P < 0.001, calculated using two-tailed unpaired *t*-test and Log-rank (Mantel–Cox) test. **E** Representative macroscopic view of tumors in ^*++*^*Oxtr* females. Scale bar: 1 mm. **F** Histology analysis of ^*++*^*Oxtr* mammary tumors. Representative images of H&E staining of mammary tumors showing major histological subtypes: **a**. Papillary carcinomas with squamous phenotype. **b**. Papillary carcinomas with mucus and lipid droplets. **c**. Medullary carcinomas. **d**. Glandular carcinomas. **e**. Poorly differentiated carcinoma. **f**. Clear cell carcinoma. Scale bar: 100 μm. Pie chart shows distribution of each subtype.

A mouse model with transgenic overexpression of OXTR under β-actin promoter (^++^*Oxtr*) was used in this study^29^. OXTR overexpression in mammary gland and brain was confirmed (Fig. S1). Seventeen out of 30 ^++^*Oxtr* females (56.6%) developed tumors in mammary gland from age of 5 to 15 months (Fig. 1A). Among the females, two (11.8%) developed more than one primary neoplasm (Fig. 1B). Tumor growth continued in size (Fig. 1C). They were removed and weighed on 30th day of onset (Fig. 1D). Tumors showed bulging surface, fleshy appearance, areas of hemorrhage, and necrosis (Fig. 1E). However, no visible lung metastases were observed (Fig. S2). No tumors were found in males.

Histological analysis was performed to classify the tumors^30^. H&E staining exhibited multifocal areas and mixed phenotypes of different morphological characteristics. Dominant phenotype for each tumor is noted. Among them, 42.1% have typical papillary patterns with small, finger-like projections. Tumors having epithelial structures with uniform and multilayered nuclei, thin stromal axes, and frond-like branching jutting into larger lumens are classified as papillary carcinomas (Fig. 1F, a, b). Squamous pathology and accumulation of keratin pearls were visible (Fig. 1F, a). Lipids in cells and mucus were detected (Fig. 1F, b). 36.8% of tumors with cord-like structures separated by thin stroma were classified as medullary carcinomas (Fig. 1F, c). 15.8% of tumors were defined as glandular carcinomas. These tumor cells have dark and irregular nuclei, form glandular-like foci or colonies of solid nests. Some tumors showed ductal areas with local invasion of tumor cells (Fig. 1F, d). In addition, some areas contain cells morphologically different from normal mammary gland cells with disorganized and irregular patterns. These are defined as poorly differentiated forms of tumors with malignant potential (Fig. 1F, e). 5.3% of tumors developed small cysts with hobnail-shaped cells or clear cytoplasm. These are classified as clear cell carcinoma (Fig. 1F, f). All results support these tumors being mammary carcinoma.

### OXTR overexpression induces mammary hyperplasia and unexpected milk production

**Fig. 2:**
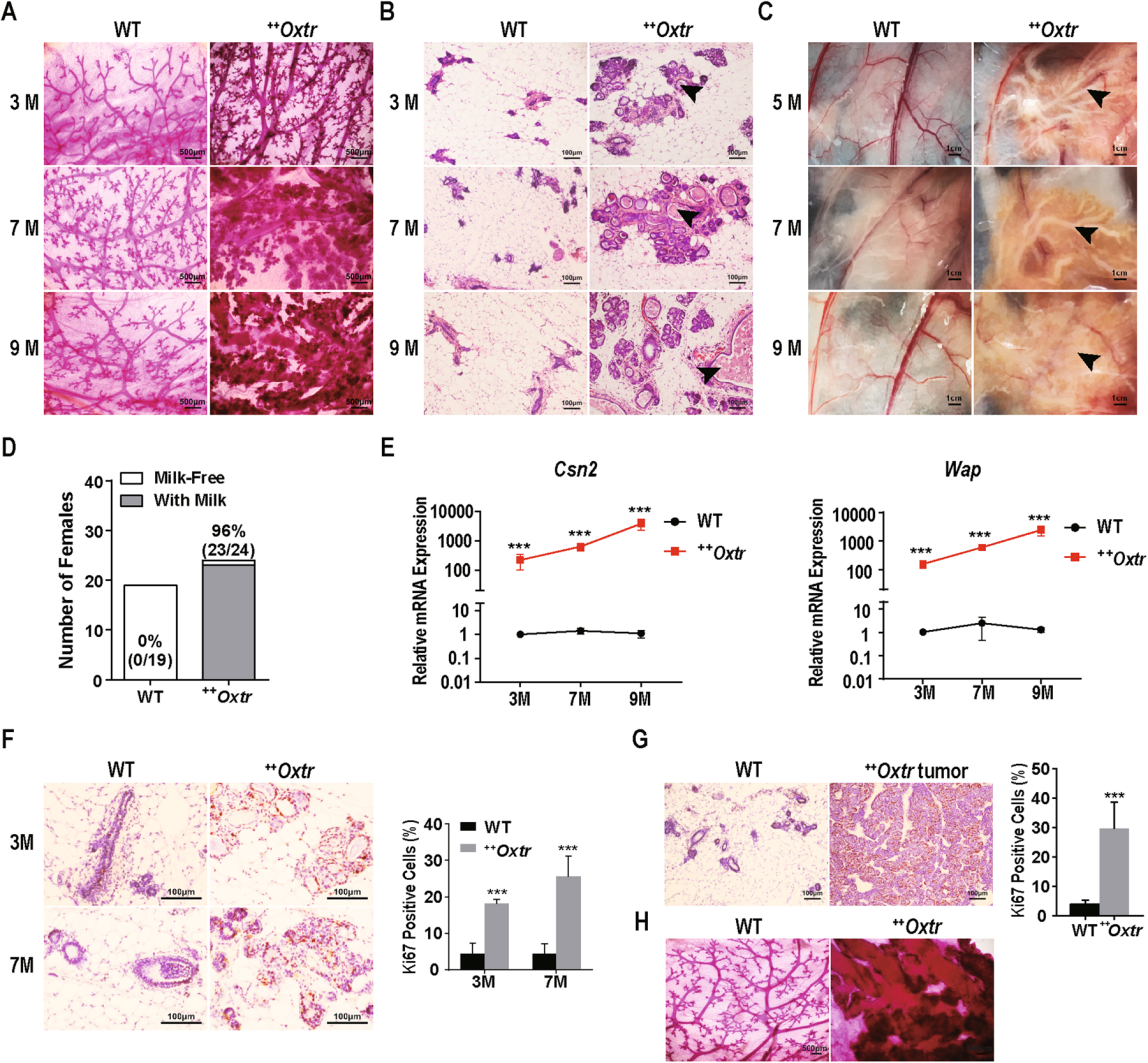
Preneoplastic mammary hyperplasia and unexpected milk production. Mammary glands (fourth pair) of ^***++***^*Oxtr* and WT females at 3, 7, and 9 months (3 M, 7 M, 9 M) were harvested. **A** Whole-mount staining of mammary gland. Scale bar: 500 μm. **B** H&E staining of mammary gland showing ducts full of proteinaceous material (Arrowhead). Scale bar: 100 μm. **C** Macroscopic images of mammary gland (third pair) with ducts full of milk (Arrowhead). Scale bar: 1 cm. **D** Milk accumulation in third mammary gland of 5-month-old WT (*n* = 19) and ^*++*^*Oxtr* females (*n* = 24). **E** Gene expression of major milk proteins *Csn2* and *Wap*, *n* = 6. **F** Immunochemistry staining of Ki67 in mammary gland. Nuclei were stained blue with hematoxylin. Scale bar: 100 μm. Quantitative immunostaining of mammary gland using Image Pro Plus, *n* = 5. **G** Ki67 immunostaining of ^*++*^*Oxtr* mammary tumor and WT mammary gland. Scale bar: 100 μm. Quantification of immunostaining using Image Pro Plus, *n* = 5. **H** Whole-mount staining of ^*++*^*Oxtr* mammary gland at tumorigenesis and corresponding WT mammary gland. Scale bar: 500 μm. Data were represented as mean ± SD. ***P < 0.001, calculated using two-tailed unpaired *t*-test.

To explore the process of mammary tumorigenesis, preneoplastic mammary gland morphology was assessed. Whole-mount staining of ^++^*Oxtr* mammary gland revealed enlarged ducts and accelerated alveoli development from age of 3 months (Fig. 2A). H&E staining showed that ducts were distended and filled with proteinaceous liquid material (Fig. 2B). Macroscopic morphology analysis revealed that the ducts were filled with milk and lasted with age (Fig. 2C). Statistically, 96% of nonpregnant ^++^*Oxtr* females showed milk accumulation (Fig. 2D). Correspondingly, qPCR results confirmed that expression of major milk protein genes *Csn2* and *Wap* were dramatically increased (221 and 157 folds at age of 3 months, and thousands of folds by 9 months, Fig. 2E). This phenotype is consistent with nipple galactorrhea in early stage of clinical breast cancer.

Immunohistochemistry (IHC) analysis of Ki67 indicated an extensive cell proliferation in ^++^*Oxtr* mammary epithelium (Fig. 2F). ^++^*Oxtr* tumors exhibited a high percentage of Ki67-positive cells (Fig. 2G). Pathological foci were readily detectable in ^++^*Oxtr* mammary gland (Fig. 2H). All results demonstrate that OXTR overexpression induces mammary hyperplasia.

### OXTR overexpression induces ERBB2^+^ mammary tumors

**Fig. 3:**
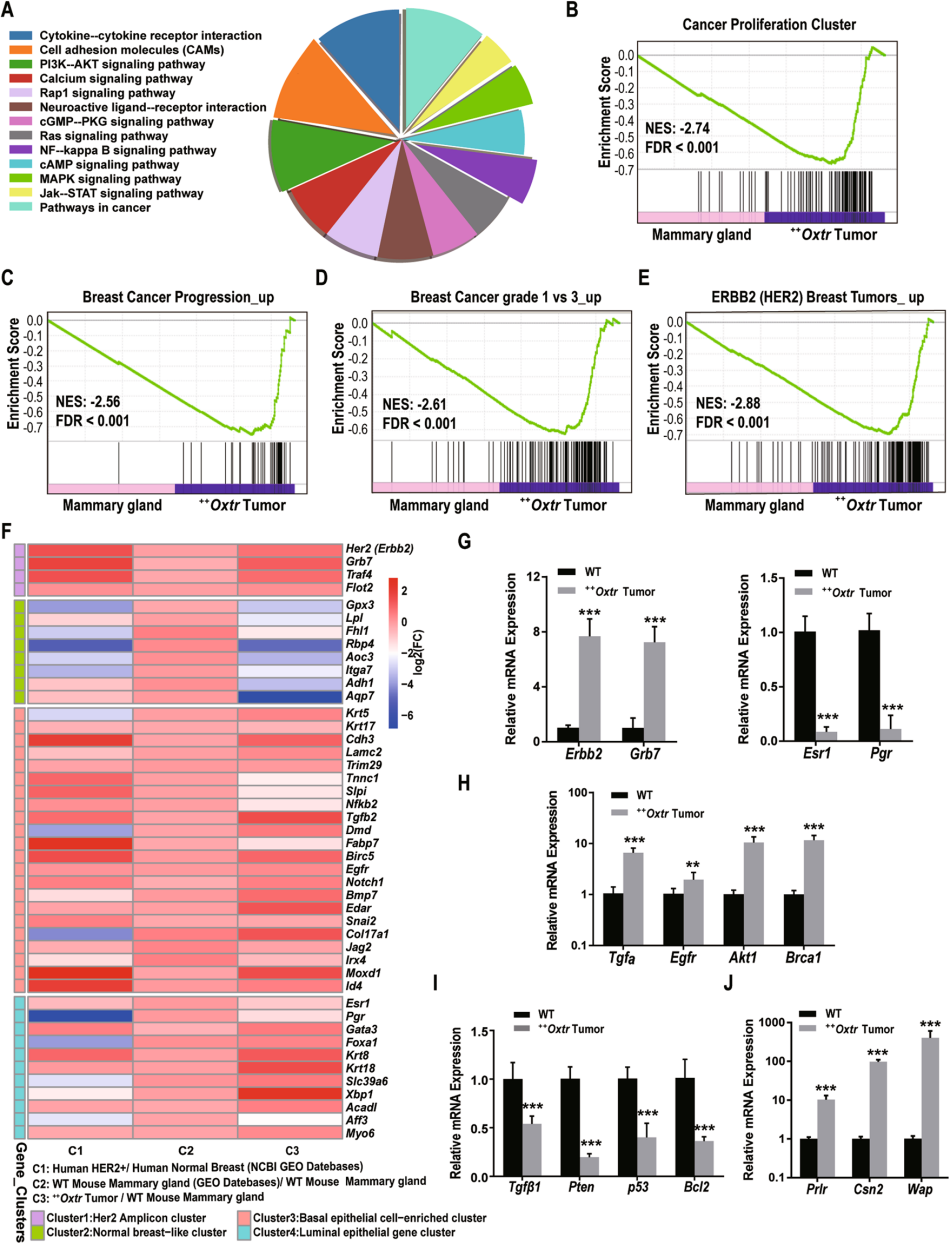
Identification of *Erbb2* positive mammary tumors by differentially expressed genes (DEGs). **A** Pie chart representation of KEGG pathway enrichment of DEGs between ^*++*^*Oxtr* tumors to WT mammary gland. **B**–**E** Gene set enrichment analysis (GSEA) showed upregulated genes of cancer proliferation cluster (**B**), breast cancer-related pathways (**C**, **D**), and ERBB2 breast cancer-related pathways (**E**) were significantly enriched in ^*++*^*Oxtr* tumors. Significance was determined by normalized enrichment score (NES) and FDR. **F** Heatmap representation of gene expression patterns in human HER2^+^ breast cancer patients to normal breast^3^ (Data from GSE61) and ^*++*^*Oxtr* tumors to WT mammary gland. **G** Validation of RNAseq results by qPCR. Gene expression of *Erbb2*, *Grb7*, *Esr1*, and *Pgr*, *n* = 6. **H** Gene expression of *Tgfa*, *Egfr*, *Akt1*, and *Brca1*, *n* = 6. **I** Gene expression of *Tgfβ1*, *Pten*, *p53*, and *Bcl2*, *n* = 6. **J** Gene expression of *Prlr*, *Csn2*, and *Wap*, *n* = 6. Data were represented as mean ± SD. **P < 0.01, ***P < 0.001, calculated using two-tailed unpaired *t*-test.

To investigate the molecular identities of these mammary tumors, RNAseq was used to analyze differentially expressed genes (DEGs) of ^++^*Oxtr* tumors against wild-type (WT) mammary gland. About 2898 DEGs were identified, 919 up and 1979 down (Fig. S3). KEGG analysis showed that DEGs were enriched in pathways of cancer, cell adhesion molecules (CAMs), cytokine–cytokine receptor interaction, PI3K-AKT, MAPK, Jak-STAT, NF-kappa B, and calcium signaling (Fig. 3A). Moreover, gene set enrichment analyses (GSEA) found that genes of cancer proliferation, breast cancer progress, and ERBB2 (HER2) breast tumors were upregulated in ^++^*Oxtr* tumors (Fig. 3B–E). Indeed, ^++^*Oxtr* tumors mimic human HER2^+^ breast cancer^3^ (Fig. 3F). ^++^*Oxtr* tumors overexpress *Erbb2* amplicon clusters including *Erbb2* (*P* < 0.0001) and *Grb7* (*P* < 0.0001) with low or no expression of *Esr1* (*P* < 0.0001) and *Pgr* (*P* < 0.0001) (Fig. 3G). IHC analysis confirmed ERBB2 overexpression in ^*++*^*Oxtr* mammary tumors. (Fig. S4). qPCR analysis confirmed RNAseq results reliable. ^++^*Oxtr* tumors showed significantly increased expression of *Tgfα* (*P* < 0.0001), *Egfr* (*P* = 0.008), *Akt1* (*P* < 0.0001), and *Brca1* (*P* < 0.0001) (Fig. 3H), and decreased expression of tumor suppressor and apoptosis genes *Tgfβ1* (*P* < 0.0001), *Pten* (*P* < 0.0001), p53 (*P* < 0.0001), and *Bcl2* (*P* < 0.0001) (Fig. 3I). Jak-STAT pathway was activated with upregulation of *Prlr* (*P* < 0.0001), *Csn2* (*P* < 0.0001), and *Wap* (*P* < 0.0001) (Fig. 3J). Gene expression pattern supports that OXTR overexpression induces ERBB2^+^ mammary tumors.

### OXTR overexpression leads to constitutive activation of PRL/p-STAT5 pathway

**Fig. 4:**
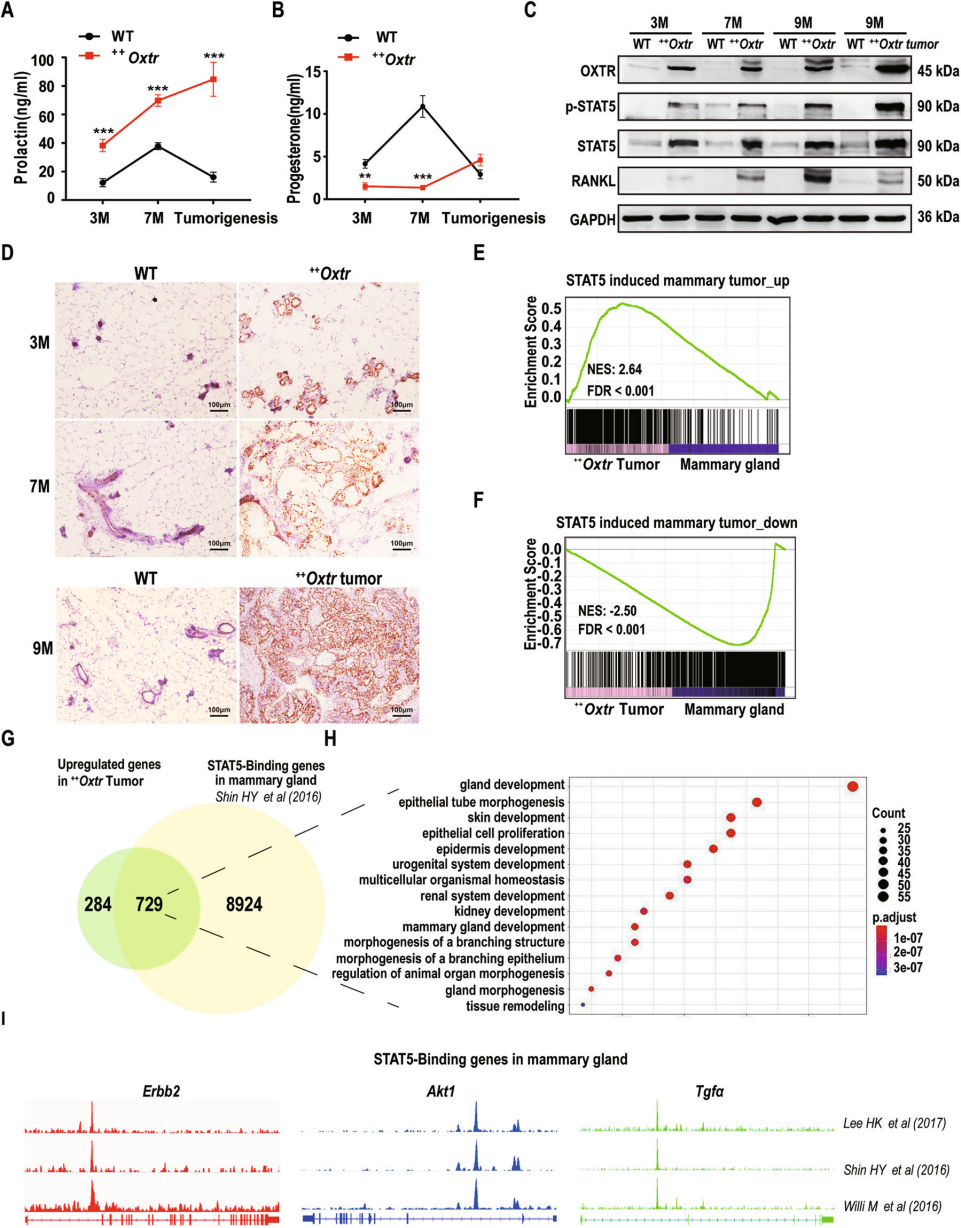
OXTR overexpression activates PRL/p-STAT5/RANKL pathway at various stages (3 M, 7 M, 9 M, Tumorigenesis). **A** Serum prolactin levels, *n* = 5. **B** Serum progesterone levels, *n* = 5. **C** Immunoblotting analysis of OXTR, p-STAT5, STAT5, and RANKL from fourth mammary gland of ^***++***^*Oxtr*, WT, and ^***++***^*Oxtr* tumors. GAPDH is the loading control. **D** Immunostaining of p-STAT5 from fourth mammary gland of ^***++***^*Oxtr*, WT, and ^***++***^*Oxtr* tumors. Nuclei were stained blue with hematoxylin. Scale bar: 100 μm. **E** GSEA plots evaluating enrichment of upregulated genes of STAT5-induced mammary tumors^31^ (Data from GSE15119 were reanalyzed) in ^***++***^*Oxtr* tumors. **F** GSEA plots evaluating enrichment of downregulated genes of STAT5-induced tumors^31^ in ^***++***^*Oxtr* tumors. **G** Venn diagram displayed the overlap between STAT5-binding genes^32^ (Data from GSE74826 were reanalyzed) and the upregulated genes in ^***++***^*Oxtr* tumors. **H** Ontology analysis of overlapping genes between OXTR-upregulated and STAT5-binding genes. **I** ChIP-seq profiles of STAT5 on *Erbb2*, *Akt1*, and *Tgfα* in mouse mammary gland^32–34^ (Data from GSE2492061, GSE74826, and GSE82275 were obtained). Data represented as mean ± SD. **P < 0.01, ***P < 0.001, calculated using two-tailed unpaired *t*-test.

To determine whether hormonal environment plays a role in OXTR-induced tumorigenesis, serum PRL, P4, estradiol, and OXT at different stages were measured. PRL levels were found to increase with age, peaked at tumorigenesis, and stayed high in ^++^*Oxtr* females (Fig. 4A). However, P4 was lower than WT (Fig. 4B). No changes were found in serum estradiol and OXT (Fig. S5). Expression of OXTR, RANKL, STAT5, and p-STAT5 in mammary gland and tumors were examined by immunoblotting. OXTR in ^++^*Oxtr* mammary gland was constantly high with highest level in tumors (Fig. 4C). RANKL was increased with age and constantly high in tumors as well while barely detectable in WT (Fig. 4C). In response to high PRL, nuclear p-STAT5 (Tyr_694_) was constantly higher with the highest level in ^++^*Oxtr* tumors (Fig. 4D). The increased nuclear p-STAT5 expressions were consistent by immunoblotting and IHC (Fig. 4C, D). Both up- and downregulated genes in STAT5-induced mammary tumors^31^ were correspondingly changed in ^++^*Oxtr* tumors (Fig. 4E, F). When comparing gene overlaps of upregulated in ^++^*Oxtr* tumors and STAT5-bound^32^, 729 genes (72%) were identified to associate with mammary gland development and epithelium cell proliferation (Fig. 4G, H). For instance, *Erbb2*, *Akt1*, and *Tgfα* upregulated in ^++^*Oxtr* tumors displayed clear enrichment of STAT5 (Fig. 4I)^32–34^. These results suggest that activation of PRL/p-STAT5/RANKL pathway is likely mediating OXTR-induced mammary tumorigenesis.

### Bromocriptine mitigates OXTR-driven hyper-mammogenesis and mammary tumor growth

**Fig. 5:**
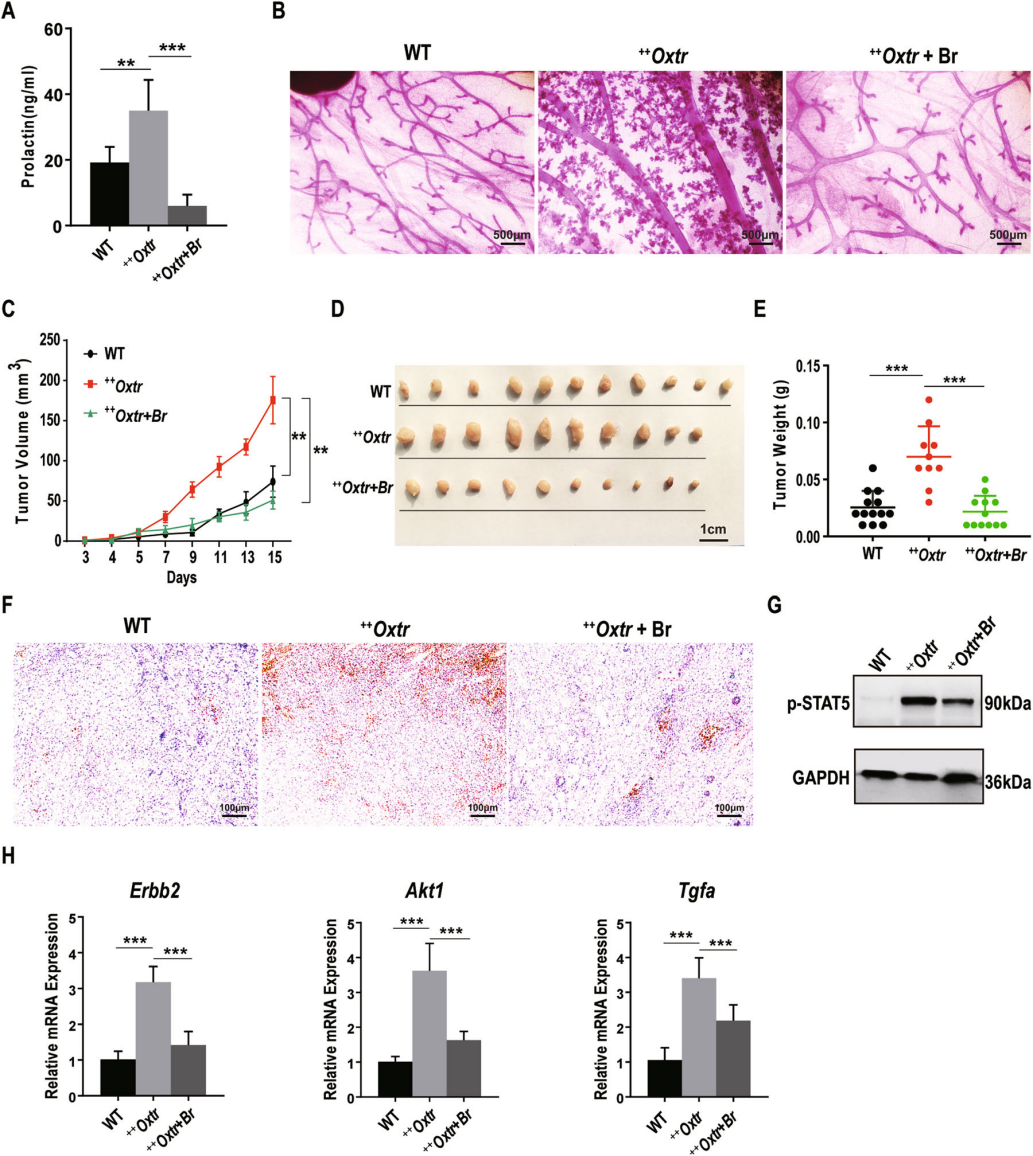
Bromocriptine treatment of ^*++*^*Oxtr* females results in compromised mammary gland development and tumor growth. After E0771 cells transplantation, ^***++***^*Oxtr* females were treated with a vehicle or 200 ug (1 mg/ml) bromocriptine (Br) for 15 days. **A** Serum prolactin (PRL) levels of WT, ^***++***^*Oxtr*, and ^***++***^*Oxtr* females with Br treatment, *n* = 8. **B** Whole-mount staining of fourth mammary glands, Scale bar: 500 μm. **C** Tumor growth (*n* = 10) by tumor volume. **D** Representative photos of tumors. **E** Tumor weights, WT (*n* = 13), ^*++*^*Oxtr* (*n* = 10), and ^***++***^*Oxtr* with Br treatment (*n* = 12). **F** p-STAT5 immunostaining of tumors. Nuclei were stained blue with hematoxylin. Scale bar: 100 μm. **G** Immunoblotting analysis of p-STAT5 of tumors. **H** Gene expression of *Erbb2*, *Akt1*, and *Tgfα* in tumors by qPCR, *n* = 6. Data were represented as mean ± SD. **P < 0.01, ***P < 0.001, calculated with one-way analysis of variance (ANOVA).

To prove that PRL signaling is mediating OXTR-induced tumorigenesis, PRL inhibitor bromocriptine (Br, 200 ug) was administered daily to ^++^*Oxtr* females transplanted with E0771, mouse breast cancer cells. Br treatment significantly decreased serum PRL levels and reversed OXTR-induced hyperprolactinemia (Fig. 5A). Whole-mount staining revealed that hyper-mammogenesis was mitigated and ^++^*Oxtr* mammary gland morphology similar to WT (Fig. 5B). Milk production in ^++^*Oxtr* mammary gland was also reduced (Fig. S6). Br significantly inhibited ^++^*Oxtr* tumor onset and growth (Fig. 5C), a significant reduction in tumor size and weight (Fig. 5D, E). IHC and immunoblotting analysis showed Br treatment diminished nuclear p-STAT5 (Fig. 5F, G). Transcription of *Erbb2*, *Akt1*, and *Tgfα*, downstream of PRL/p-STAT5 signaling, was inhibited (Fig. 5H). Lapatinib (a tyrosine kinase inhibitor targeting ERBB2, 100 ug/g) was orally administered twice daily for 15 days from E0771 injection. However, no significant difference was found in tumor growth between treated and untreated ^++^*Oxtr* females (Fig. S7). These results support that OXTR induces mammary tumorigenesis through PRL/p-STAT5 pathway.

### OXTR overexpression creates a microenvironment that promotes mammary tumor growth and metastasis

**Fig. 6:**
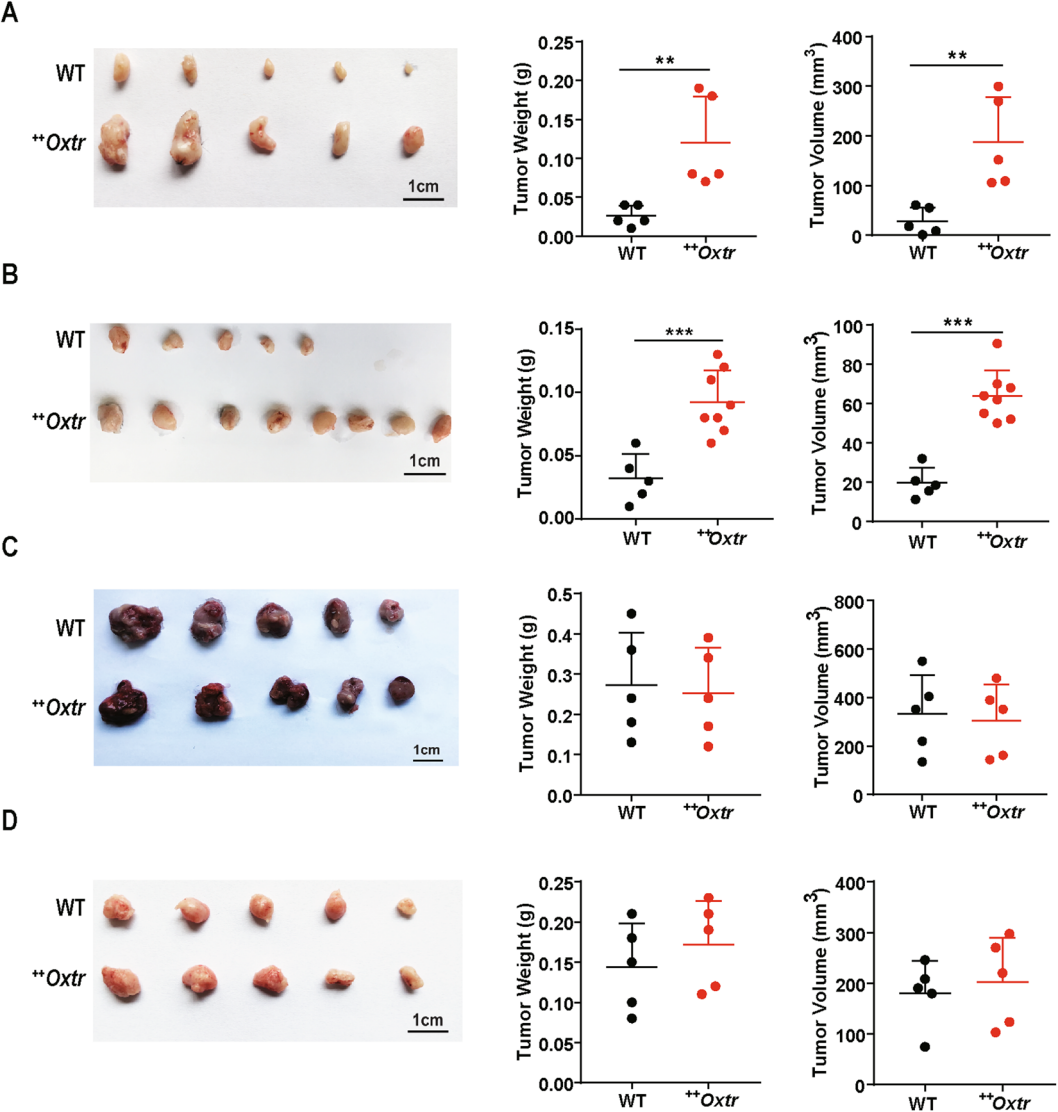
OXTR overexpression promotes mammary tumor growth, not melanoma or cervical tumor. For tumor growth analysis, ^++^*Oxtr* tumor fragment, E0771, B16, or U14 cells were orthotopically transplanted into fourth mammary gland of 3-month-old WT and ^++^*Oxtr* females. Tumors were analyzed on day 15 after transplantation. **A** Representative photos of mammary tumors from ^++^*Oxtr* tumor fragment, Scale bar: 1 cm. Quantitative analysis of mammary tumor weights (*n* = 5) and volumes (*n* = 5). **B** Representative photos of mammary tumors from E0771 cells, Scale bar: 1 cm. Quantitative analysis of mammary tumor weights (*n* = 5 from WT and *n* = 8 from ^++^*Oxtr*) and volumes (*n* = 5 from WT and *n* = 8 from ^++^*Oxtr*). **C** Representative photos of melanoma tumors from B16 cells, Scale bar: 1 cm. Quantitative analysis of melanoma tumor weights (*n* = 5) and volumes (*n* = 5). **D** Representative photos of cervical tumors from U14 cells, Scale bar: 1 cm. Quantitative analysis of cervical tumor weights (*n* = 5) and volumes (*n* = 5). Data were represented as mean ± SD. **P < 0.01, ***P < 0.001, calculated using two-tailed unpaired *t*-test.

To assess whether altered hormonal environment creates a driving force for tumorigenesis, 1 mm^3^ piece of ^++^*Oxtr* tumor, E0771, B16 (melanoma cells), or U14 (cervical tumor cells) were orthotopically transplanted into fourth mammary gland of ^++^*Oxtr* and WT females. After 15 days, mice were checked for tumor growth and tumor weight. Tumors from ^++^*Oxtr* tumor fragment or E0771 cells were much larger in ^*++*^*Oxtr* females than those in WT (Fig. 6A, B). However, no difference was detectable in B16 or U14 tumor growth between ^++^*Oxtr* and WT (Fig. 6C, D). OXTR overexpression may have created a microenvironment that specifically promotes mammary tumor growth.

To assess whether OXTR-induced microenvironment can drive metastasis as well, E0771 and B16 cells were injected through tail vein. Larger numbers of visible metastases were readily detectable in ^++^*Oxtr* lungs from E0771 (Fig. S8A, B) with marked increase of metastatic foci (Fig. S8C). However, no significant difference was detected from B16-injected mice (Fig. S8D). Results suggest that OXTR-induced microenvironment can promote mammary-specific tumor growth and metastasis but not melanoma tumors.

## Discussion

**Fig. 7:**
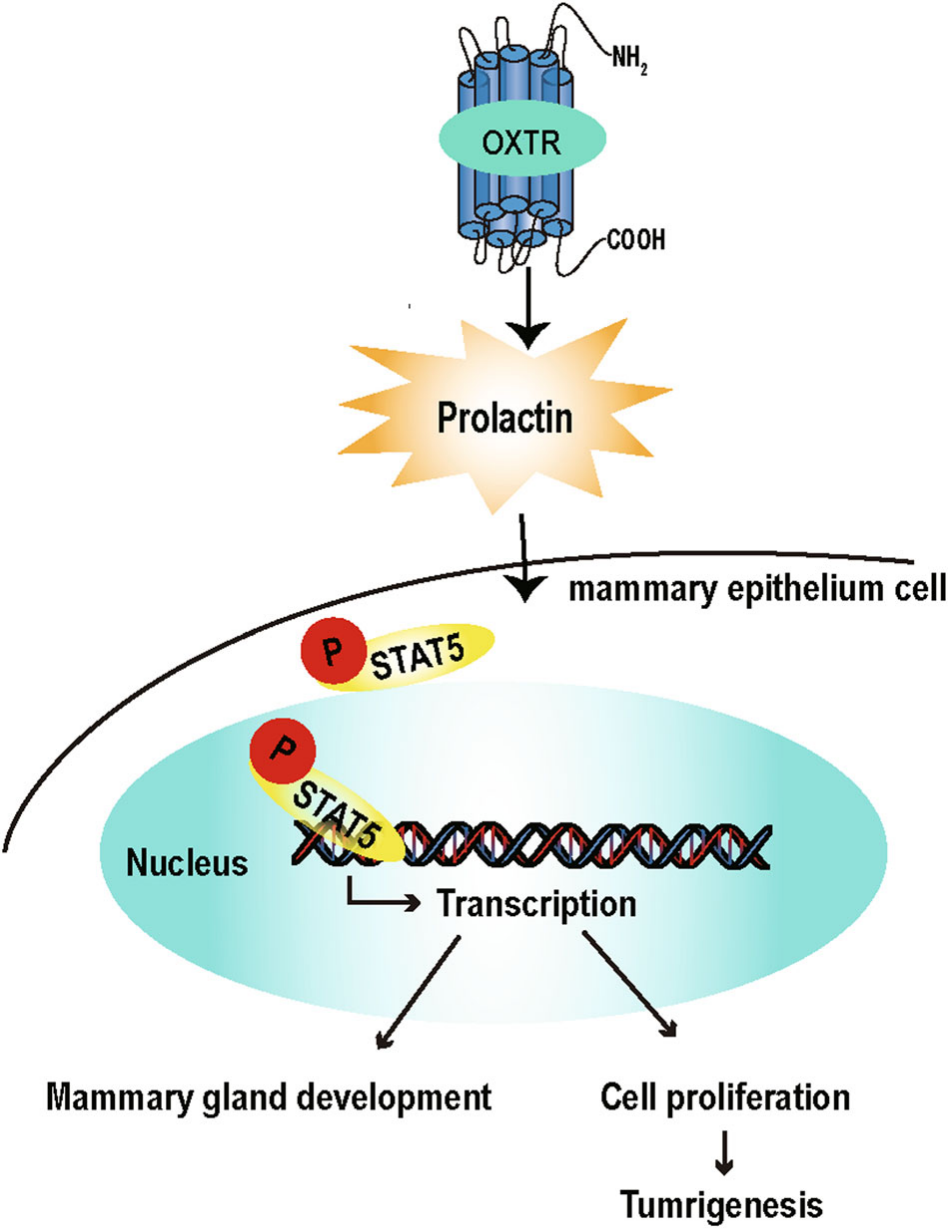
Role model of OXTR in mammary tumorigenesis. OXTR overexpression leads to increased prolactin secretion in ^++^*Oxtr* females. Prolactin induces phosphorylation and nuclear translocation of p-STAT5 to promote transcription of genes responsible for cell proliferation and milk proteins (*Csn2* and *Wap*). Excessive proliferation of mammary epithelium induces tumorigenesis.

OXTR overexpression induces dramatic PRL secretion and STAT5 phosphorylation. Nuclear translocation of p-STAT5 leads to increased transcription of mammary epithelial proliferation-related genes, accelerated mammary gland development (unexpected milk secretion), and tumorigenesis (Fig. 7). OXTR induces hormonal changes and creates a mammary gland-specific environment that promotes mammary tumor growth.

Mouse models of mammary tumorigenesis have been established to mimic various subtypes of human breast cancers^8^. Overexpression of HER2 is associated with metastasis and poor prognosis^35,36^. Our study shows that 57% of ^++^*Oxtr* females develop ERBB2^+^ mammary tumors with change of PI3K-AKT, MAPK, Jak-STAT, and NF-kappa B pathways, similar to HER2^+^ breast cancer. High HER2 is accompanied by activation of PI3K/AKT and MAPK pathways, promoting cellular proliferation and survival^37^. ^++^*Oxtr* tumors are morphologically mixed with papillary and medullary carcinoma that are invasive and highly malignant. OXTR-induced hyperprolactinemia, unexpected milk production (nipple discharge), and mammary hyperplasia are all early characteristics of human breast cancer. ^++^*Oxtr* mouse should be an ideal model for HER2^+^ drug screening and testing.

OXTR overexpression induced high PRL. The excessive PRL secretion leads to accelerated mammogenesis and tumorigenesis. Studies using mouse models lacking either PRL (*Prl*^−/−^) or activated STAT5 have confirmed the role of PRL/p-STAT5 signaling in mammary gland development^38,39^. In response to PRL, p-STAT5 translocates to nucleus and activates target gene transcription^40^. We have identified that 72% of upregulated genes in ^++^*Oxtr* tumors are targets of STAT5 and function in mammary gland development and epithelium cell proliferation. STAT5-targeted genes *Erbb2*, *Akt1*, *Tgfα*, *Csn2*, and *Wap* were all upregulated in ^++^*Oxtr* tumors. These rationalize the early symptoms of preneoplasia including mammary hyperplasia, unexpected milk production, and *Erbb2*^*+*^ mammary tumorigenesis in ^++^*Oxtr* females. Our study demonstrates that PRL/p-STAT5 signaling mediates OXTR-induced mammary tumorigenesis. PRL stimulates breast cancer cell proliferation through HER2 expression^41^. Hyperprolactinemia increases risk of breast cancer^42^. High blood PRL is associated with poor prognosis and low survival with metastatic breast cancer^43^. *Prl* overexpression in mouse mammary gland or transplanted pituitary glands induces mammary carcinomas in aged females^44,45^. Incidence of neoplasms in these females with moderate latency is similar to that of ^++^*Oxtr* females. These reports all support our hypothesis that OXTR-induced *ERBB2*^*+*^ mammary tumors through increased PRL secretion. Br, an inhibitor of PRL/p-STAT5 pathway, can effectively block OXTR-induced PRL secretion, ERBB2 expression, hyper-mammogenesis, and tumorigenesis. The result confirms the role of OXTR through PRL/p-STAT5. The relationship of OXTR, hyperprolactinemia, and ERBB2 expression in breast cancer is established in this study. Moreover, study has shown that metastatic disease-related hyperprolactinemia is significantly more frequent in HER2^+^ patients^46^, suggesting PRL may stimulate HER2 expression. PRL may be a potential marker for diagnosis of HER2^+^ breast cancer. Lapatinib, the inhibitor of ERBB2, cannot compromise ^++^*Oxtr* tumor growth. This result suggests that ERBB2 may not be the sole mediator of PRL/p-STAT5-stimulated breast cancer cell proliferation. Study has shown attempts to interfere with HER2 alone have failed to yield an effective treatment^47^.

RANKL is regulated by both P4 and PRL^48,49^. OXTR overexpression leads to increased PRL secretion but downregulates P4. PRL/p-STAT5 axis increased RANKL expression despite low P4, suggesting limited role of P4 in OXTR-induced tumorigenesis. Our results show that OXTR overexpression induces mammary hyperplasia and tumorigenesis by activation of PRL/p-STAT5/RANKL axis.

Our results indicate that OXTR-induced hormonal environment promotes mammary tumorigenesis exclusively. OXTR expression was detected in human breast cancer cells T47D, MCF7, ZR-75-30, and MDA-MB-231 (Fig. S9). Whether these tumor cells can secrete PRL needs to be determined. In addition, contribution of mammary/brain OXTR overexpression on PRL secretion requires evaluation. We previously reported that mammary OXTR is not a major player in abnormal mammary gland development^29^. OXTR expression in brain can respond to exogenous OXT and stimulates PRL release from pituitary lactotroph^50,51^. We assume that OXTR overexpression in brain may be the major source of PRL secretion in ^++^*Oxtr* mice. Neuron- and mammary gland-specific overexpression of OXTR may further shed light on mammary tumorigenesis.

In conclusion, we have found OXTR overexpression induces ERBB2^+^ mammary tumors through activation of PRL/p-STAT5 pathway. The activation creates an environment that promotes mammary gland-specific tumor growth. *Oxtr* is a novel oncogene and a potential new drug target for HER2^+^ breast cancer. PRL is an important marker for HER2-tumor diagnosis and drug target for HER2^+^ breast cancer. In addition, Br is an effective antitumor drug for OXTR/PRL-driven HER2^+^ breast cancer.

## Materials and methods

### Materials and reagents

All general chemicals and reagents were purchased from Sigma, USA and Takara, China.

### Cell lines

Medullary tumor cell line E0771 (RRID: CVCL_GR23), melanoma cell line B16 (CVCL_0157), and cervical cancer cell line U14 (CVCL_9U56) with C57/BL6J genetic background were from ATCC. E0771 and B16/U14 cells were maintained in RPMI 1640 and DMEM supplemented with 10% fetal bovine serum (Gibco) at 37 °C, 100% humidity, and 5% CO_2_. Human breast cancer cell lines T47D (CVCL_0553), MCF7 (CVCL_0031), ZR-75-30 (CVCL_1661), and MDA-MB-231 (CVCL_0062) were gifted from Jilin People’s Hospital and cultured as described^52^.

### Animals

All animal studies were performed in accordance with Guide for Care and Use of Laboratory Animals from National Institutes of Health and approved by the Ethics Committee of Shenyang Medical College (SYYXY2018030101). Generation of β-actin-*Oxtr* (^*++*^*Oxtr*) mice (RRID: MGI: 6314370) by us was described previously^29^. Age-matched WT littermates were used as controls. All animals were switched to C57/BL6J background and maintained under pathogen-free conditions at 21 ± 1 °C, 50 ± 20% relative humidity, with free access to food and water, and 12:12 h light/dark cycle. Mice were anesthetized with 1% pentobarbital natrium (10 mg/kg) intraperitoneally before euthanizing.

### Genotyping

^*++*^*Oxtr* mice were genotyped by PCR^53^ using primers Forward (1785–1806): AATGCCCTGGCTCACAAATAC and Reverse (2240–2263): GGGACAGCTATGACTGGGAGTAG in polyA regions of pCAGGS. Tail tips were digested with GNTK buffer at 55 °C overnight^54^. The lysates were boiled for 15 min as DNA templates. PCR conditions are 2 min at 94 °C, followed by 30 cycles of 30 s at 94 °C, 30 s at 57 °C, and 1 min at 72 °C. Final extension was 10 min at 72 °C.

### Histology and IHC analysis

Mammary glands and tumors were fixed in 4% paraformaldehyde (PFA) for 24 h. Fixed tissues were dehydrated, embedded in paraffin, sectioned at 5 μm, and stained with hematoxylin and eosin (H&E)^55^. For immunostaining^56^, antigen was heat-retrieved for 15 min in EDTA (pH 8.0) or Citrate (pH 6.0), and incubated with primary antibodies: rabbit anti-OXTR (ab181077, Abcam, 1:500), rabbit anti-Ki67 (D3B5, CST, 1:500), rabbit anti-ERBB2/HER2 (D8F12, CST, 1:400), and rabbit anti-Phosoho-STAT5 (9359 s, CST, 1:600) at 4 °C overnight. Slides were incubated with HRP-conjugated anti-rabbit IgG (8114 P, CST, USA) for 30 min at room temperature. Signal was revealed with DAB (CST, USA). Finally, slides were counterstained with hematoxylin and examined under an Olympus IX71 microscope.

### Whole-mount staining

The fourth inguinal mammary glands were dissected and spread on glass slides. After fixation with Carnoy solution, glands were rehydrated gradually through a series of diluted ethanol and immersed in carmine aluminum solution^57^ at room temperature overnight. Glands were dehydrated through serial ethanol baths and cleared in xylene.

### RNAseq and analysis

Libraries were constructed from WT mammary glands and ^*++*^*Oxtr* tumors, and sequenced using an Illumina Hiseq platform. Low-quality reads were removed^58^. Clean reads were mapped to mouse genome sequence using TopHat2^59^. Results were presented as fragments per kilobase of transcript per million of mapped reads (FPKMs)^60^. *Q* value < 0.05 and |log2 (fold change)| >1 were used as threshold for significantly different expression by Cuff diff version 2.0.0^61^. Gene ontology (GO) analysis was performed using Gorilla and estimated by hypergeometric test using custom R scripts. Significance (*p* value) was adjusted by false discovery rate (FDR)^62^. GO terms with *q* value < 0.05 were regarded as significantly enriched. The enrichment scores were calculated using Gene Set Enrichment Analysis (GSEA) as described before^63^. OmicShare small tools2 was used to obtain heatmaps. Threshold parameters were set as no rows and column clusters. The GeneVenn online tool was used to create Venn diagrams of gene lists.

### Quantitative real-time PCR (qPCR)

Total RNA was purified from mouse tissues using Trizol reagent (Takara). One microgram RNA was reverse transcribed with Prime Script cDNA Synthesis Kit (Takara). The cDNAs were used for PCR with SYBR Green Mix (Takara) following the manufacturer’s instruction. Relative expression level was normalized to 18 S ribosomal RNA and calculated using 2^−ΔΔCT^ value method. PCR primers are listed in Table S1.

### Immunoblotting

Cells or mouse tissues were homogenized in RIPA buffer. A 40 μg of total protein was separated on 10% SDS-PAGE and transferred to PVDF membrane^64^. The membranes were incubated with primary antibodies rabbit anti-OXTR (ab181077, 1:5000), rabbit anti-STAT5 (94205 s, 1:1000), rabbit anti-Phosoho-STAT5 (9359 s, 1:1000), goat anti-RANKL (AF462, R&D Systems, 1:2000), and rabbit anti-GAPDH (AP0063, Bioworld, 1:10,000) at 4 °C overnight. Membranes were incubated with secondary antibodies HRP-conjugated donkey anti-rabbit IgG (GE Healthcare, 1:3000) and HRP-conjugated rabbit anti-goat IgG (BS30503, Bioworld, 1:10,000) for 1 h at room temperature. Amersham^™^ ECL^™^ (GE Healthcare) was used to detect signals. GAPDH was served as a loading control.

### Tumor tissue/cell transplantation

A 1 mm^3^ piece of tumor fragment, E0771, B16, or U14 cells (5 × 10^6^) were orthotopically transplanted into fourth mammary gland of 3-month-old WT or ^++^*Oxtr* virgin females. Tumor sizes were measured using digital calipers and volume was calculated as ½ (length × width^2^). Tumors from E0771, B16, and U14 cells were removed after 15 days of injection. For tail vein injection, E0771 and B16 cells (1 × 10^6^) were suspended in PBS before injection. Metastatic lesions from E0771 or B16 were examined in 4 weeks or day 15 after injection.

### ELISA assay

Serum samples were prepared by clotting for 30 min at room temperature and centrifuged at 400x*g* for 10 min. ELISA assays were performed according to the manufacturer’s instruction. Sensitivities of mouse P4 (Cat DEV9988, DEMEDITEC Diagnostics GmbH), mouse PRL (Cat AB100736, Abcam), 17β-Estradiol (Cat ADI-900-174, Enzo), and OXT (Cat ADI-900-153, Enzo) are 0.04 ng/mL, 30 pg/mL, 14 pg/mL, and 15 pg/mL. Absorbance was measured using microplate reader (SpectraMax, Molecular Device, USA).

### Bromocriptine and Lapatinib treatment

Br (Sigma, USA) was dissolved in sterile saline (0.9% NaCl) to a final concentration of 1 mg/ml. After tumor cells injection, mice were treated daily with 200 μg Br subcutaneously for 15 days. Control WT and ^++^*Oxtr* females were treated in parallel with saline.

Lapatinib (Selleck, USA) was dissolved in solvents (2% DMSO + 30% PEG300 + 5% Tween 80 + ddH2O) individually and in order. Oral administrations of Lapatinib (100 ug/g) with ^++^*Oxtr* females were twice daily for 15 days from E0771 injection.

### Statistical analysis

All data were presented as means ± SD. *P* value was calculated with unpaired two-tailed Student’s *t*-tests to compare two groups, one-way ANOVA to compare more than three groups, and log-rank (Mantel–Cox) test for survival analysis. Asterisks denote statistically significant differences (*P < 0.05; **P < 0.01; ***P < 0.001).

## Supplementary information

Supplementary Figure Legends

Fig. S1

Fig. S2

Fig. S3

Fig. S4

Fig. S5

Fig. S6

Fig. S7

Fig. S8

Fig. S9

Table S1

## Data Availability

Data reported has been deposited in Gene Expression Omnibus (GEO) database (accession number: PRJNA542227).
