# Supplementary material for: Oxytocin receptor induces mammary tumorigenesis through prolactin/p-STAT5 pathway
Source: Cell Death Dis. 2021 Jun 7;12(6):588. doi: 10.1038/s41419-021-03849-8 (PMC8184747; doi:10.1038/s41419-021-03849-8)
Supplement: Supplementary file 1 — Supplementary Figure Legends [file 41419_2021_3849_MOESM1_ESM.docx]

**Supplementary Figure Legends**

**Fig. S1 Overexpression of OXTR in mammary glands and brain of *^++^Oxtr* mice. (A)** Immunochemistry staining of OXTR from the 4^th^ mammary gland of 3-month-old ***^++^****Oxtr,* WT females. Nuclei were counter-stained blue with hematoxylin. Scale bar: 100μm. **(B)** Immunoblotting analysis of OXTR in brain of 3-month-old ***^++^****Oxtr,* WT females. GAPDH was served as a loading control.

**Fig. S2 Spontaneous lung** **metastasis analysis.** After 30 days of tumor onset, the females were examined for metastatic lesions. Representative H&E staining of lungs of ***^++^****Oxtr* and WT females. Scale bar: 200μm.

**Fig. S3 Volcano map of differentially expressed genes (DEGs) in mammary tumors.** DEGs were identified with criterion of Fold Change ≥2, adjusted P<0.05 and gene count >2 between *^++^Oxtr* tumors and WT mammary gland. Green dots represent 1979 down-regulated genes, red dots represent 919 up-regulated genes, and black dots represent non-differentially expressed genes.

**Fig.S4 Spontaneous mammary tumors of ^++^*Oxtr* females with high ERBB2.** ERBB2 immunostaining of WT mammary gland, WT tumor, **^++^*Oxtr*** tumors. Nuclei were stained blue with hematoxylin. Scale bar: 50μm.

**Fig. S5 Measurement of serum hormones. (A)** Serum estradiol levels in ***^++^****Oxtr* and WT females at age of 3M and 7M, n=5 for each time point. **(B)** Serum oxytocin levels in ***^++^****Oxtr* and WT females at age of 3M and 7M, n=5 for each time point. Data are represented as mean ±SD, calculated using two-tailed unpaired t test.

**Fig. S6 Bromocriptine effect on mammary gland development. *^++^****Oxtr* females were treated with a vehicle or 200ug (1mg/ml) bromocriptine (Br) for 15 days. **(A)** Macroscopic images of 3^th^ mammary glands. Scale bar: 1cm. Quantitative real time PCR (RT-PCR) analysis of *Csn2* **(B)** and *Wap* **(C)** expression in 4^th^ mammary glands, n=6. Data are represented as mean ±SD. ***P <0.001, calculated with one-way analysis of variance (ANOVA).

**Fig. S7 Lapatinib treatment has no effect on tumor growth of *^++^Oxtr* females injected with E0771 cells.** After E0771 cells transplantation, ***^++^****Oxtr* females were treated with a vehicle or Lapatinib (100ug/g) for 15 days. **(A)** Tumor growth of WT, ***^++^****Oxtr* females and ***^++^****Oxtr* females with Lapatinib treatment by tumor volume, (n=5). **(B)** Representative photos of tumors on day 15 after transplantation. **(C)** Tumor weights (n=5) on day 15 after transplantation.

**Fig. S8 Metastasis analysis though tail vein-injection.** For metastasis analysis, E0771 cells were tail vein injected into 3-month-old WT and ^++^*Oxtr* females and growth nodules in lung were identified 4 weeks later. **(A)** Representative photos of lungs. Arrowheads point to metastatic nodules in lungs. **(B)** Total numbers of metastatic nodules, n=5. **(C)** Representative H&E staining of lungs. Arrowheads point to metastatic foci in lungs. **(D)** B16 cells were tail vein-injected into 3-month-old ^++^*Oxtr* and WT females. Two weeks later, mice were examined for metastatic lesions. Growth nodules in lung were recorded, n=5. Data are represented as mean ±SD, *0.01<P<0.05, calculated using two-tailed unpaired t test.

**Fig. S9 OXTR expression in human breast cancer cells.** Immunoblotting of OXTR in human breast cancer cells. GAPDH is served as a loading control.
