# Supplementary figures and images for: Oxytocin receptor induces mammary tumorigenesis through prolactin/p-STAT5 pathway
Source: Cell Death Dis. 2021 Jun 7;12(6):588. doi: 10.1038/s41419-021-03849-8 (PMC8184747; doi:10.1038/s41419-021-03849-8)

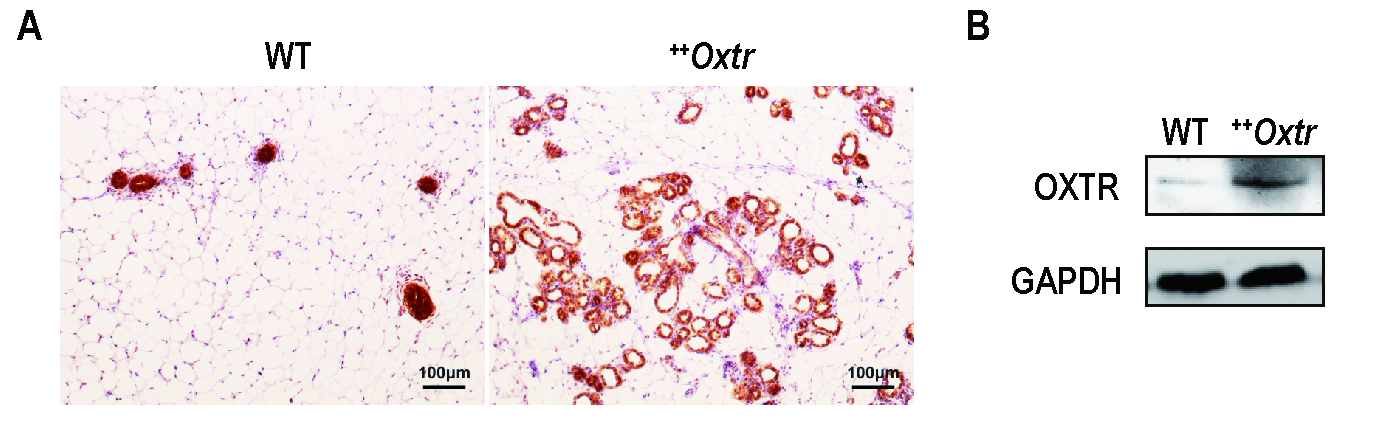

Supplement: Supplementary file 2 — Fig. S1 [file 41419_2021_3849_MOESM2_ESM.tif]

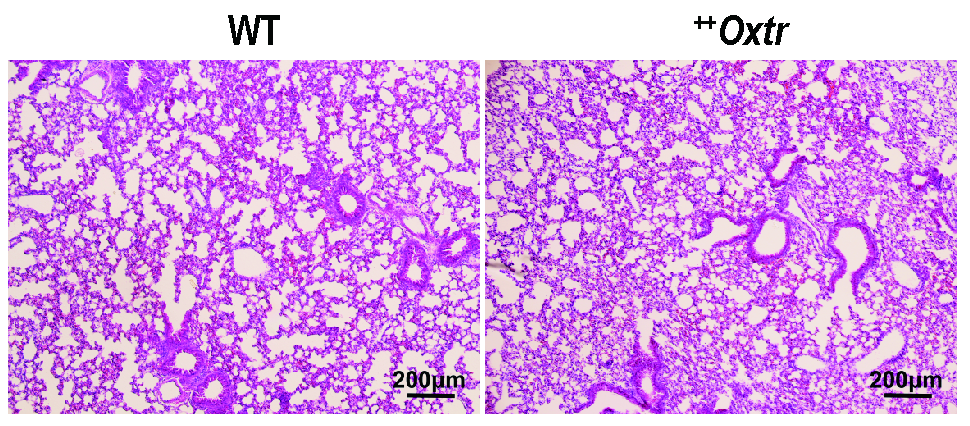

Supplement: Supplementary file 3 — Fig. S2 [file 41419_2021_3849_MOESM3_ESM.tif]

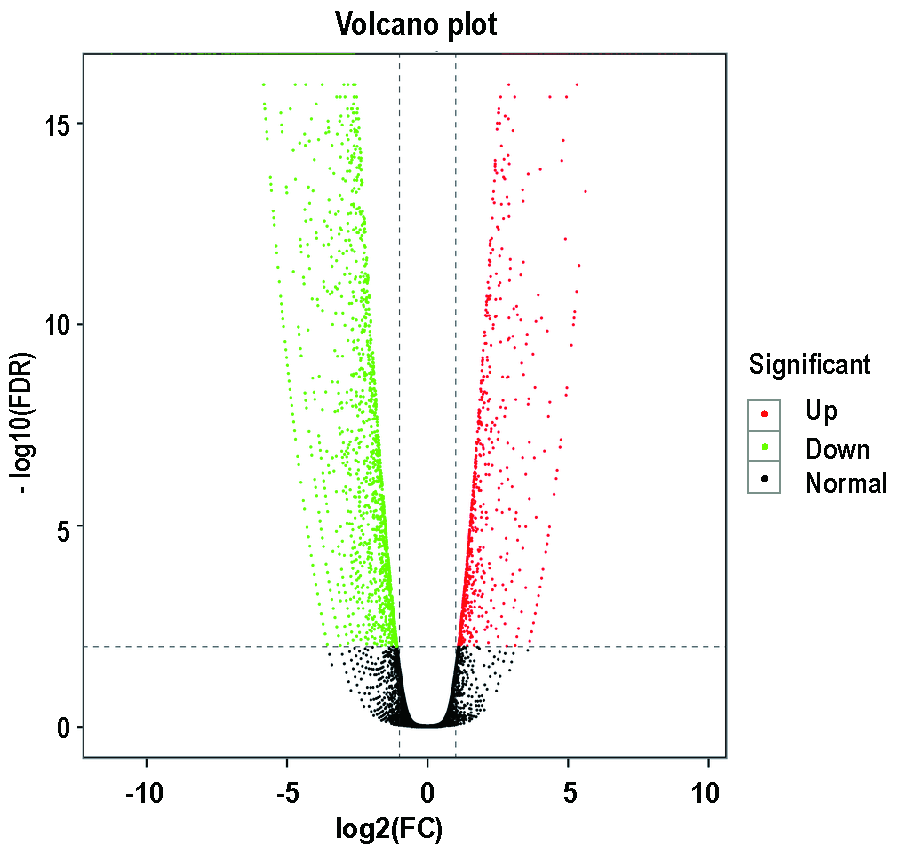

Supplement: Supplementary file 4 — Fig. S3 [file 41419_2021_3849_MOESM4_ESM.tif]

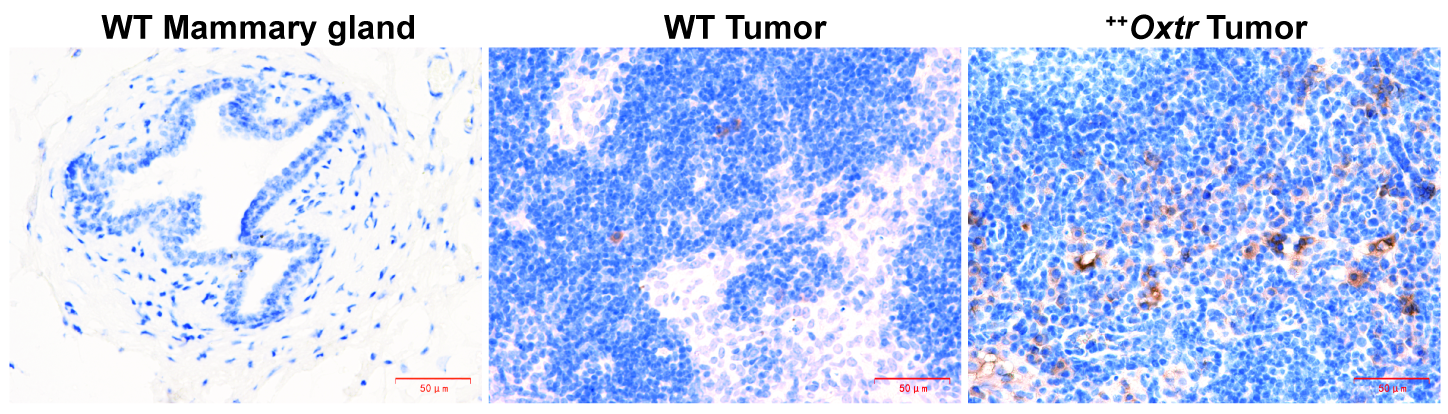

Supplement: Supplementary file 5 — Fig. S4 [file 41419_2021_3849_MOESM5_ESM.tif]

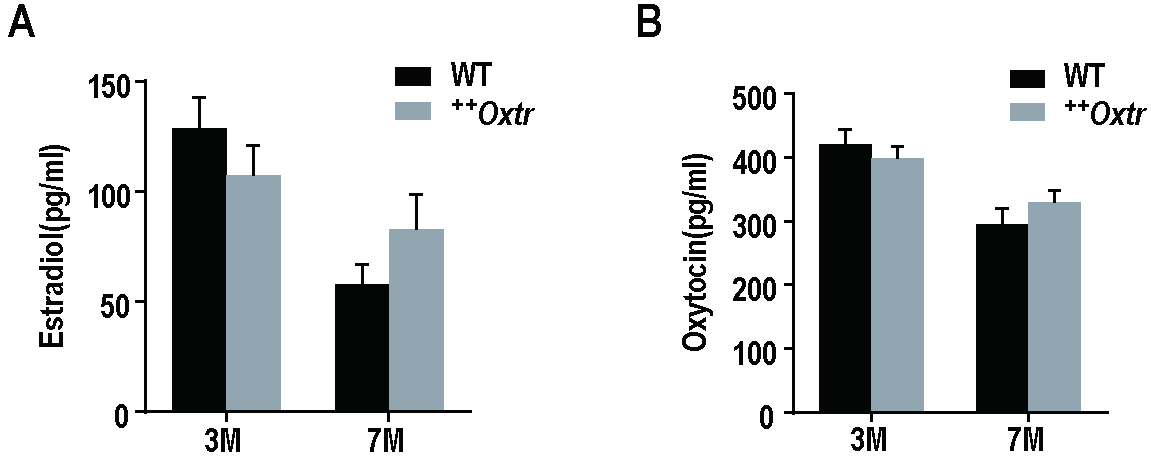

Supplement: Supplementary file 6 — Fig. S5 [file 41419_2021_3849_MOESM6_ESM.tif]

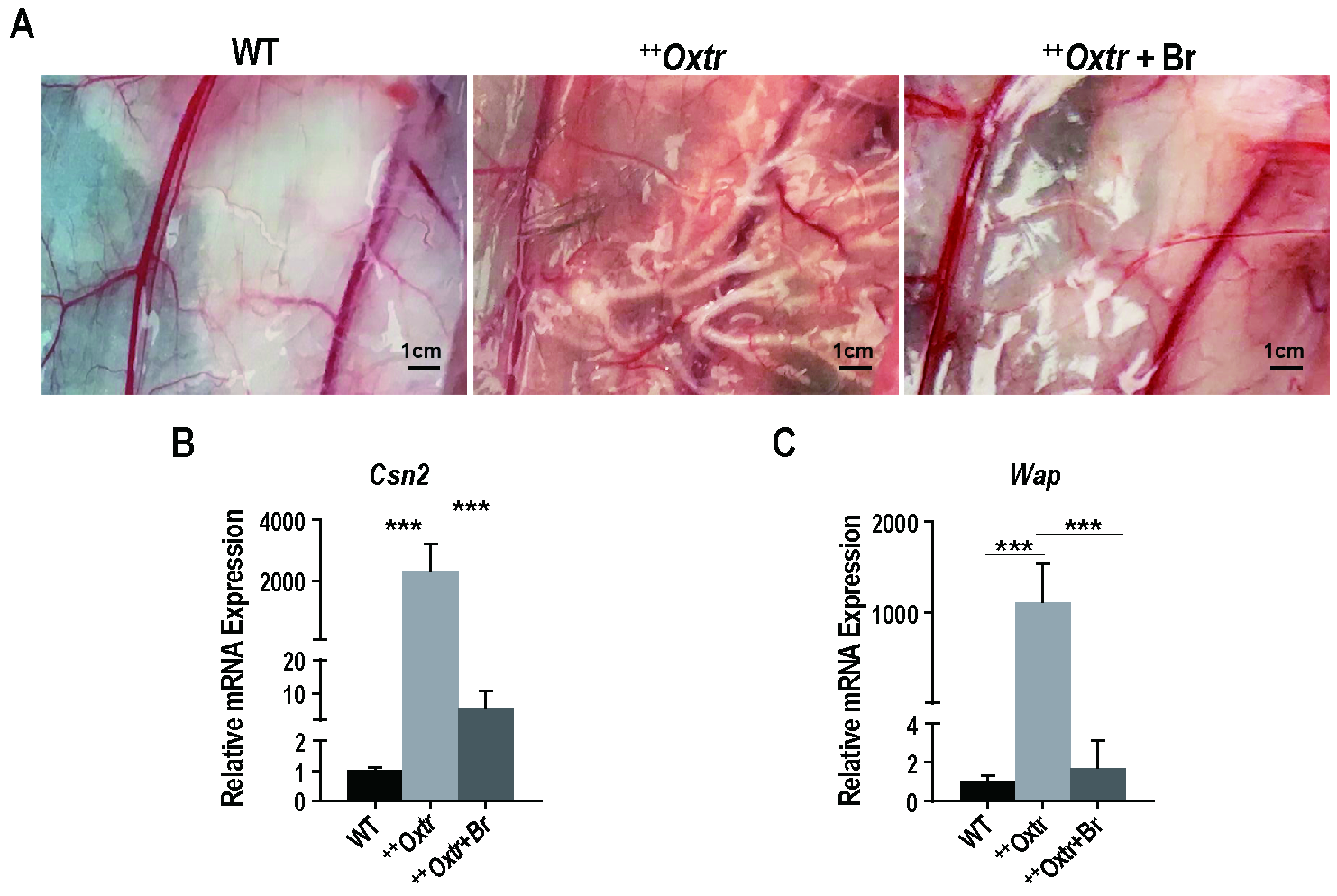

Supplement: Supplementary file 7 — Fig. S6 [file 41419_2021_3849_MOESM7_ESM.tif]

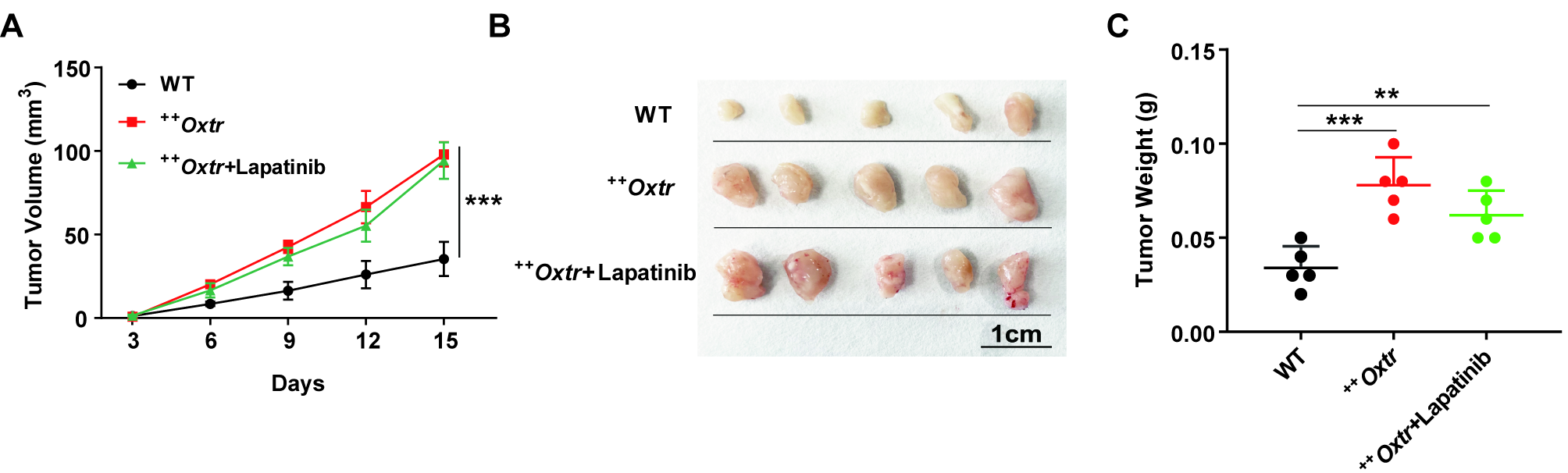

Supplement: Supplementary file 8 — Fig. S7 [file 41419_2021_3849_MOESM8_ESM.tif]

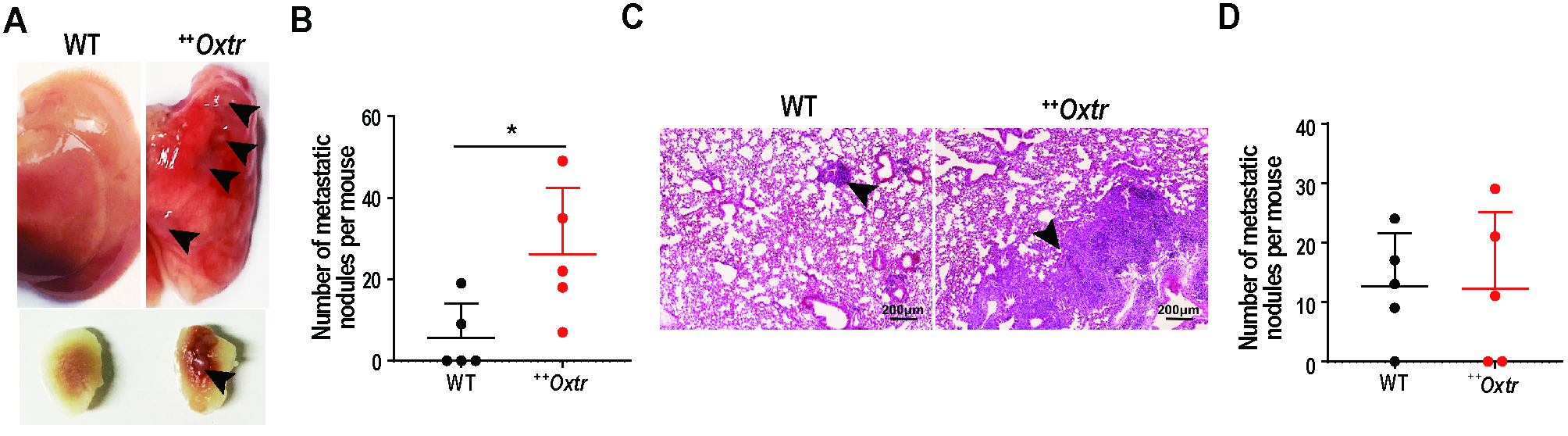

Supplement: Supplementary file 9 — Fig. S8 [file 41419_2021_3849_MOESM9_ESM.tif]

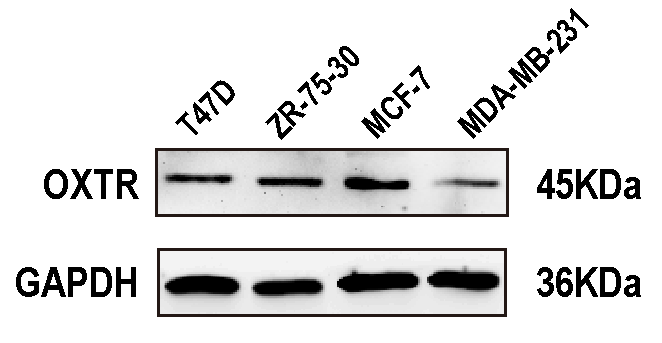

Supplement: Supplementary file 10 — Fig. S9 [file 41419_2021_3849_MOESM10_ESM.tif]
