# Supplementary material for: Oxytocin receptor induces mammary tumorigenesis through prolactin/p-STAT5 pathway
Source: Cell Death Dis. 2021 Jun 7;12(6):588. doi: 10.1038/s41419-021-03849-8 (PMC8184747; doi:10.1038/s41419-021-03849-8)
Supplement: Supplementary file 11 — Table S1 [file 41419_2021_3849_MOESM11_ESM.docx]

**Table S1.** Primer sequences used for qPCR

| Target gene | Primer sequences | Accession number |
| --- | --- | --- |
| 18s -Forward | CGCCGCTAGAGGTGAAATTC | NR_003278.3 |
| 18s -Reverse | CGAACCTCCGACTTTCGTTCT |  |
| *Her2*- Forward | CCTGCCCCTACAACTACCTCTCCA | [NM_001003817.1](https://www.ncbi.nlm.nih.gov/nuccore/NM_001003817.1) |
| *Her2*- Reverse | ATTTCTCACACCGCTGTGTTCCGT |  |
| *Grb7-* Forward | CAGAGGAACCCACAGGGCTTTGT | [NM_010346.2](https://www.ncbi.nlm.nih.gov/nuccore/NM_010346.2) |
| *Grb7-* Reverse | AACCTTCATCTTCGCTTGGCAAAAT |  |
| *Esr1-* Forward | TGTCCAGCAGTAACGAGAAAGG | [NM_001302531.1](https://www.ncbi.nlm.nih.gov/nuccore/NM_001302531.1) |
| *Esr1-* Reverse | TGGTAGCCAGAGGCATAGTCAT |  |
| *Pgr* -Forward | CCGAGTTATGAGAACCCTTGACG | NM_008829.2 |
| *Pgr* -Reverse | CGGGACCAGTTGAATTTCTTGAT |  |
| *Tgfβ1-* Forward | GTGGACCGCAACAACGCCATCTA | [NM_011577.2](https://www.ncbi.nlm.nih.gov/nuccore/NM_011577.2) |
| *Tgfβ1-* Reverse | CACTGCTTCCCGAATGTCTGACGTA |  |
| *Pten-* Forward | CATTTGCAGTATAGAGCGTGCAGAT | [NM_008960.2](https://www.ncbi.nlm.nih.gov/nuccore/NM_008960.2) |
| *Pten-* Reverse | ATTTGGAGAGAAGTATCGGTTGGC |  |
| *p53-* Forward | CCGCCGACCTATCCTTACCATCAT | [NM_001127233.1](https://www.ncbi.nlm.nih.gov/nuccore/NM_001127233.1) |
| *p53-* Reverse | CAGGCACAAACACGAACCTCAAAG |  |
| *Bcl2-* Forward | GGTGGTGGAGGAACTCTTCAGGGAT | [NM_009741.5](https://www.ncbi.nlm.nih.gov/nuccore/NM_009741.5) |
| *Bcl2-* Reverse | GTTGACGCTCTCCACACACATGACC |  |
| *Tgfα-* Forward | TGTGTGCCAGGCTCTGGAGAACAGC | [NM_031199.4](https://www.ncbi.nlm.nih.gov/nuccore/NM_031199.4) |
| *Tgfα-* Reverse | GTGTGGGAATCTGGGCACTTGTTGA |  |
| *Egfr-* Forward | TGGGCCCTGTCGCAAAGTTTGTAAT | [NM_007912.4](https://www.ncbi.nlm.nih.gov/nuccore/NM_007912.4) |
| *Egfr-* Reverse | AGGATGTGAAGGTCCCCGCTGAT |  |
| *Akt1-* Forward | AGAAGAACGTGGTGTACCGGGACCT | [NM_001165894.1](https://www.ncbi.nlm.nih.gov/nuccore/NM_001165894.1) |
| *Akt1-* Reverse | CAGCCCGAAGTCCGTTATCTTGATG |  |
| *Brca1-* Forward | CTTCTCCAAACCAGTAGAGGATAAT | [NM_009764.3](https://www.ncbi.nlm.nih.gov/nuccore/NM_009764.3) |
| *Brca1-* Reverse | TATAATTTCAGTCACATGGTTCAGG |  |
| *Prlr* -Forward | ACCATGGATACTGGAGTAGATGGGG | NM_001253781.1 |
| *Prlr* -Reverse | AAACAGATGACAGCAGAGAGAACGG |  |
| *Csn2*-Forward | TCCTTAAAGCTAAAGCCACCATCC | NM_001286020.1 |
| *Csn2*-Reverse | AGAGTTTATGAGGCGGAGCACAGT |  |
| *Wap* -Forward | ATCATCTGCCAAACCAACGAGG | NM_011709.5 |
| *Wap* -Reverse | AGCCAGCTTTCGGAACACCAAT |  |

The primers were designed to span an exon-exon junction using Primer Premier 6 following the principle (product size: 80-130bp, melt temperature: 63-68°C, GC content: 40%-60%). The melt curves and the standard curves were used to validate primer specificity and efficiency.
